# Wrist Position Sense in Two Dimensions: Between-Hand Symmetry and Anisotropic Accuracy Across the Space

**DOI:** 10.3389/fnhum.2021.662768

**Published:** 2021-04-22

**Authors:** Giulia A. Albanese, Michael W. R. Holmes, Francesca Marini, Pietro Morasso, Jacopo Zenzeri

**Affiliations:** ^1^Department of Robotics, Brain and Cognitive Sciences, Istituto Italiano di Tecnologia, Genova, Italy; ^2^Department of Informatics, Bioengineering, Robotics and Systems Engineering (DIBRIS), University of Genoa, Genoa, Italy; ^3^Faculty of Applied Health Sciences, Brock University, St. Catharines, ON, Canada; ^4^MathWorks, Natick, MA, United States

**Keywords:** proprioception, robotic assessment, directional error, range of motion, wrist position sense

## Abstract

A deep investigation of proprioceptive processes is necessary to understand the relationship between sensory afferent inputs and motor outcomes. In this work, we investigate whether and how perception of wrist position is influenced by the direction along which the movement occurs. Most previous studies have tested Joint Position Sense (JPS) through 1 degree of freedom (DoF) wrist movements, such as flexion/extension (FE) or radial/ulnar deviation (RUD). However, the wrist joint has 3-DoF and many activities of daily living produce combined movements, requiring at least 2-DoF wrist coordination. For this reason, in this study, target positions involved movement directions that combined wrist flexion or extension with radial or ulnar deviation. The chosen task was a robot-aided Joint Position Matching (JPM), in which blindfolded participants actively reproduced a previously passively assumed target joint configuration. The JPM performance of 20 healthy participants was quantified through measures of accuracy and precision, in terms of both perceived target direction and distance along each direction of movement. Twelve different directions of movement were selected and both hands tested. The left and right hand led to comparable results, both target extents and directions were differently perceived according to the target direction on the FE/RUD space. Moreover, during 2-DoF combined movements, subjects’ perception of directions was impaired when compared to 1-DoF target movements. In summary, our results showed that human perception of wrist position on the FE/RUD space is symmetric between hands but not isotropic among movement directions.

## Introduction

Proprioception is the multimodal perceptual process that allows humans to maintain global awareness of their body position during active or passive movements. Sensory signals coming from receptors located in joints, muscles, tendons and skin, are encoded biomechanically and transmitted to the Central Nervous System (CNS), which carries out multi-sensory association, allowing individuals to be aware of their body posture, position in space, movements and applied forces. Particularly, different receptors provide different facets of proprioceptive information: Golgi-type endings detect forces and tensile strain at the limit of the range of motion (Oksuz et al., 2018); Ruffini-type mechanoreceptors, typically present in wrist ligaments, are likely to be involved in the perception of both wrist positions and motions (Hagert et al., 2005); Pacini corpuscles, more rarely identified, are sensitive to the onset, offset and velocity of motions, suggesting that this information is probably less important for sensorimotor processes; muscle spindles, located in intrafusal muscle fibers, provide information about muscle length and speed of muscle stretch. Notably, far from maximum angles of joint motions, muscle spindles were demonstrated to be the main receptors responsible for providing information about the sense of position, while Golgi tendon organs, joint and skin receptors are all less involved (Proske and Gandevia, 2012).

Proprioception is a necessary sensory process to accomplish most activities of daily living. Proprioception allows us to control our limbs without the need of visual feedback and is crucial to motor planning (Touzalin-Chretien et al., 2010) and updating our body schema (Morasso et al., 2015). More specifically, for understanding the role played by proprioception in the control of movement (Riemann and Lephart, 2002), we should consider that proprioceptive deprivation does not preclude gross motor functions (Schabrun and Hillier, 2009), though these subjects do have significant motor deficits (Goble et al., 2012). Generally, proprioceptive deficits are associated with both neurological and orthopedic conditions: in particular, proprioception is altered in both stroke (Langhorne et al., 2009) and Parkinson’s Disease (Rickards and Cody, 1997), and also in peripheral sensory neuropathies (Rothwell et al., 1982), cervical dystonia (Avanzino et al., 2020), or injuries to ligaments, joint capsules and muscles (Barrack et al., 1989). In order to improve motor recovery and maximize the effectiveness of rehabilitation protocols for these clinical populations, a deep investigation of proprioception is necessary to clarify how these sensory afferents are related and contribute to the motor outcome (Masia et al., 2009a; De Santis et al., 2015; Zabihhosseinian et al., 2015). Despite this, the influence of both neural mechanisms (Marini et al., 2019) and intrinsic wrist mechanical properties (Albanese et al., 2019; Falzarano et al., 2020) on proprioceptive processes has still been poorly investigated.

One specific proprioceptive function, the Joint Position Sense (JPS), plays a critical role in many learning paradigms, influencing therehabilitative outcome (Schmidt and Lee, 1988). JPS is the ability toreproduce a defined joint angle, usually evaluated through the JointPosition Matching (JPM) Test. In this work, we focused on the wrist, a joint essential for fine control, manipulation and haptic perception during most activities of daily living. A set of studies have recently focused on measuring the wrist position sense through a robotic device, separately for the three degrees of freedom (DoF) of the wrist (Cappello et al., 2015; Contu et al., 2015; Marini et al., 2016b, 2017b): flexion/extension (FE), radial/ulnar deviation (RUD), and pronation/supination (PS). On the other hand, it cannot be ignored that most activities of daily living require complex wrist gestures, involving the accurate coordination of at least 2-DoF (Brigstocke et al., 2014). However, no study has yet to investigate how wrist position is perceived during multi-DoF joint movements and whether JPS changes between the dominant and non-dominant hand. For this reason, in the present work, we evaluated the wrist position sense across the FE/RUD space, by means of combined movements of flexion or extension with a radial or ulnar deviation. The aim was to inspect how wrist movements along different target directions are proprioceptively perceived in the two hands, investigating whether the coordination of 2-DoF could affect accuracy and precision in the perception of wrist position.

## Materials and Methods

### Experimental Setup and Participants

**FIGURE 1:**
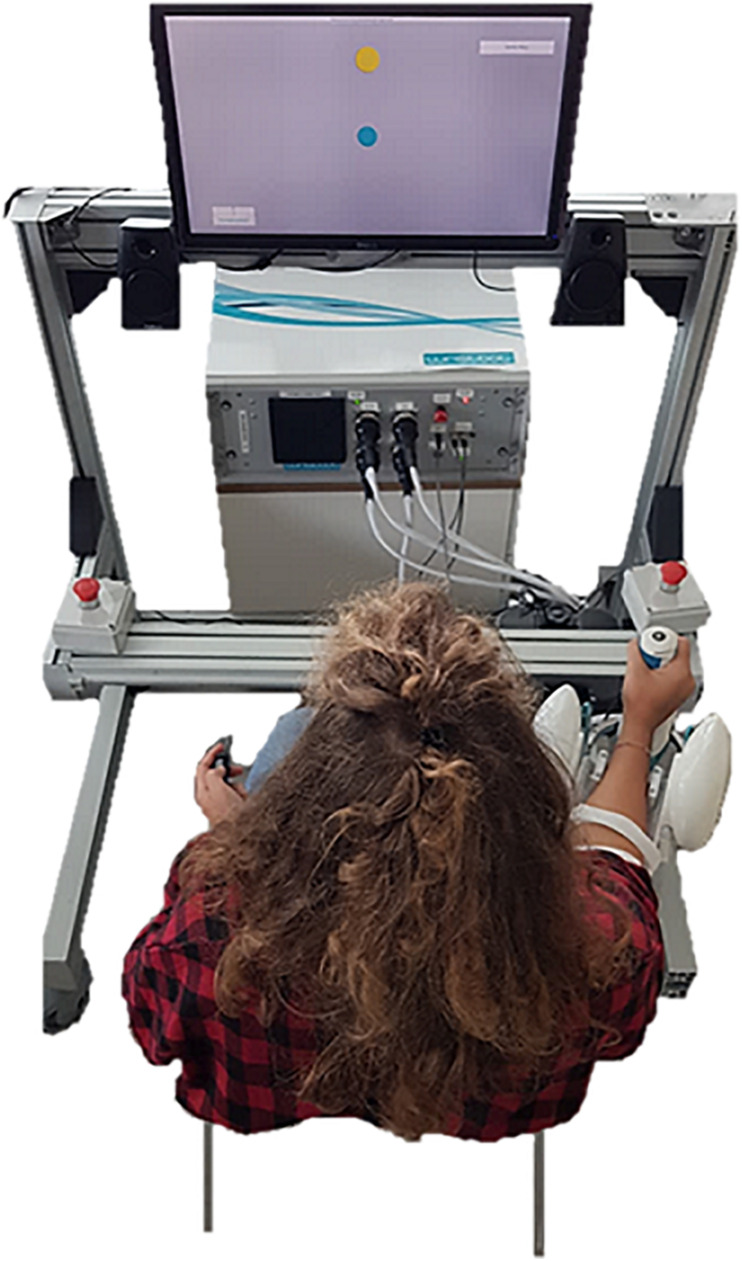
Experimental setup: subjects sat in front of the screen, with their right arm strapped to the forearm support, holding the grip sensor mounted on the handle. The screen was central and 80 cm from the eyes of the subject. The robot was rotated 25° externally to assure a more natural posture during the task. The subject, not blindfolded yet, is performing the active ROM assessment.

Twenty subjects (8 males, 27.2 ± 1.8 years) with no history of neurological disorders enrolled in this study. According to the Edinburgh Handedness Inventory (Oldfield, 1971) all subjects were right-handed. Experiments were carried out at the Motor Learning, Assistive and Robotics Rehabilitation Laboratory at the Istituto Italiano di Tecnologia (Genoa, Italy). The study was approved by the local ethical committee of Liguria Region (n.222REG2015), in accordance with the Declaration of Helsinki. Subjects gave their informed consent to participate in the study. During the testing protocol, subjects sat on a chair in front of a screen, holding a button in their left hand and a fully backdrivable manipulandum in their right hand (Figure 1). The robotic device used for the test, the WRISTBOT (Masia et al., 2009b; Iandolo et al., 2019), is a three-DoF manipulandum that allows wrist movements with three DoF, namely flexion/extension (FE), radial/ulnar deviation (RUD) and pronation/supination (PS), compatible with the full range of motion (ROM) of human subjects. The device is equipped with four brushless motors, able to deliver torques to manipulate the wrist joint, compensate weight and guarantee low inertia. Angular displacements on the three axes are measured by high-resolution incremental encoders at a 100 Hz sample rate, while an integrated virtual reality environment provides visual feedback to the user. The forearm support was provided with an adjustable constraining structure and Velcro bands to ensure absence of forearm movements and perfect alignment of the wrist with the axes of the mechanical structure. The handle of the robot was wrapped with a custom-made soft sensor able to measure the applied pressure, with the aim of identifying changes in the grip force exerted by subjects during the task. Specifically, the sensor consisted of an expanded polyurethane covered by a material with nickel and copper wires, and the electric resistance could be modified according to grip force changes. The change in resistance was converted to a voltage decrease through an external electric circuit and sent to the electronic board of the robot. Finally, a proper calibration procedure was executed in order to directly relate a change in voltage to force measured in Newton.

### Experimental Protocol

The testing protocol consisted of the assessment of two components of wrist functionality, namely the active ROM and the JPS, the latter tested through a JPM task.

#### Active ROM

**FIGURE 2:**
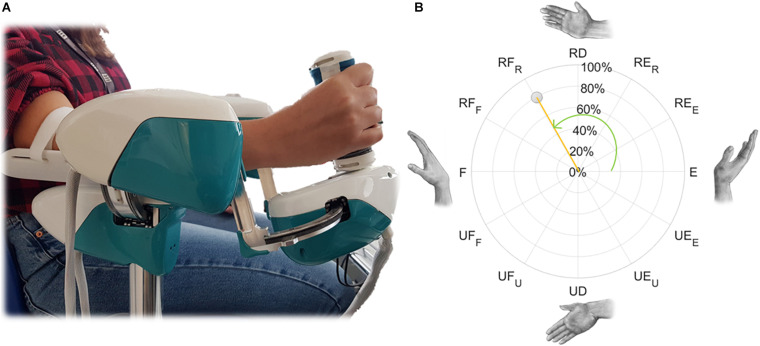
Example of a wrist movement for the right hand **(A)** and its polar representation in the FE-RUD space **(B)**, described by *normalized magnitude* (orange) and *direction* (green). *Direction* is expressed as an angle: 0° for extension movements in both hands and this value increases counterclockwise for the right hand and clockwise for the left hand. *Normalized magnitude*, expressed as percentage, is null in the neutral position.

After an acoustic sound, a target appeared on the screen and subjects were instructed to move the freely rotating robotic device from the neutral position (zero-degree rotation on each DoF) in the direction of the target, as far as they could go. Then, the hand was passively brought back to the neutral position by the robot and a new target was presented to the subject. Targets were located at the farthest point allowed by the robot, along 12 equally spaced directions in the FE-RUD space (Figure 2). Movement directions included movements in the space described by these two DoF (FE/RUD), along the four main directions of movement on those planes (Flexion F, Extension E, Radial RD, and Ulnar deviation UD), but also along combined directions involving both DoF. Targets were randomized and presented twice. The outcome measure we were interested in was the maximum ROM in degrees measured by the encoders for each target, i.e., the measure of the maximum wrist rotation along that specific direction.

#### Joint Position Matching

**FIGURE 3:**
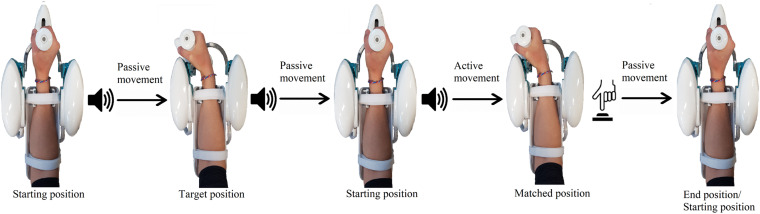
Example of a single trial, involving a flexion movement. During passive movements, the robot moves the hand of the subject, while, during the active phase, the subject moves actively to reproduce the previously assumed joint configuration. The same sound was played for both movements starting from the neutral position, while a different sound was played when the robot kept the subject on the target position for 3 s. When the active matching was completed, the subject pushed a button to notify the end.

Subjects were blindfolded for this task and were required to maintain a natural grip force on the handle. Each trial consisted of three phases (Figure 3):

1. *Passive phase*: from the anatomical neutral position, a first auditory signal was played to notify the robot starting to move the hand of the subject along a specified direction at 10 deg/s, with a minimum jerk velocity profile. When the target position was reached, a different auditory cue was presented, and the robot held the hand steady for 3 s. Successively, the hand was passively moved back to the neutral position.
2. *Matching phase*: after a notification sound, subjects were instructed to actively reproduce the configuration assumed in the first phase and specify the end of the matching movement by pressing the button with the left hand. During this phase, the robotic system did not provide force, apart from gravity compensation, and was entirely moved by the subject, without constraints on the two DoF (FE/RUD).
3. *Returning phase*: once the active matching phase was terminated, the robot passively moved the hand back to the neutral configuration, prior to starting a new trial.

Targets were located along the 12 directions whose active ROM was previously assessed. Each target set, composed by 12 targets, was repeated 6 times, corresponding to a total of 72 trials. According to the real-time grip sensor measurements, recorded vocal cues were sent to the subject warning to hold less or more (“release” or “hold” messages) to assure a comparable grip force on the handle across target sets. Since subjects were requested to maintain a natural grip force during the experiment, warning messages were sent after those trials whose measured grip fell out of the maximum-minimum grip range assessed during the first target set. In order to make both subjects and target directions comparable among themselves, the target magnitude was set to 80% of the subject assessed ROM along each direction (Marini et al., 2016b). To maintain the subject’s attention and avoid the effects of fatigue on proprioceptive acuity (Mugnosso et al., 2018, 2019), 2 min of rest were provided after three target sets.

### Data Analysis

Joint rotations data measured by each encoder were filtered with a sixth-order Savitzky-Golay low-pass filter (10 Hz cut-off frequency). Wrist positions in the JPM task, recorded in terms of single-DoF rotations (α_FE_,α_RUD_), were evaluated considering two components of movement, *normalized magnitude* and *direction*. They corresponded to the two polar components of the vector linking the neutral wrist position to its final target/matched position. The *normalized magnitude* represented the extent of the wrist angular rotation and was expressed as a percentage of the maximum ROM (ROM %) reached by each subject along each direction (Eq. 1). Angular *directions*, expressed in degrees, were computed as shown in Eq. (2).

(1) $$\mathrm{normalized}\,\mathrm{magnitude}=100*\frac{\sqrt{\alpha_{\text{FE}}^{2}+\alpha_{\text{RUD}}^{2}}}{\text{ROM}}$$

(2) $$\text{direction}=\text{arctan}\,\left(\frac{\alpha_{\text{RUD}}}{\alpha_{\text{FE}}}\right)$$

Performance was evaluated through widely used measures of accuracy and precision, computed for each target position (Matching Error, Variability and Error Bias) (Marini et al., 2016a,b). Since movements were not constrained to the target direction, subjects were free to commit directional errors during matching movements. For this reason, differently from previous studies, measures of performance were computed considering independently *normalized magnitude* and *direction* components.

Specifically, the Matching Error (ME) is a measure of accuracy and it represents the mean difference across *N* = 6 repetitions between the target **t** and the position **m** matched by the subject in each trial **i** (Eq. 3). These differences were computed considering as **t** and **m** each of the two above-mentioned components separately: ME_mag_ was related to differences in terms of extent, while ME_dir_ to directional errors.

(3) $$\mathrm{ME}=\frac{\sum_{i=1}^{\text{N}}\left|t-m_{\text{i}}\right|}{N}$$

Analogously, Variability (VAR) provides a measure of the consistency across *N* = 6 repetitions for each subject (Eq. 4) and was computed in terms of both *normalized magnitude* (VAR_mag_) and *direction* (VAR_dir_).

(4) VAR=std⁢(m)

Finally, Error Bias (EB) was computed as the mean signed difference across *N* = 6 repetition between the target **t** and the position **m** matched by the subject in each trial **i** (Eq. 5). Through EB, it is possible to recognize whether the subject, moving along his own direction of movement, underestimated or overestimated the correct target extent. For this reason, EB was computed only for the component related to the length of movement, i.e., its *normalized magnitude*.

(5) $$\text{EB}=\frac{\sum_{i=1}^{N}\left(t-m_{\text{i}}\right)}{\text{N}}$$

### Statistical Analysis

Normality of data was checked using a Shapiro-Wilk Test. Since data were not normally distributed, a non-parametric one-way ANOVA (Kruskal-Wallis) was chosen to evaluate differences of the main indicators, namely ROM, Matching Error, Variability and Error Bias. Data were inspected using different factors hands, planes, directions and DoF. In case of significance (*p* < 0.05), *post-hoc* pairwise comparisons using Dwass-Steel-Critchlow-Fligner (DSCF) were performed to investigate where differences occurred. Two One-Sided Tests (TOST) were used to test between-hand equivalence (Cohen’s d equivalence bounds Δ = [-0.5, 0.5]). Statistical analysis was conducted using the Jamovi Statistical Data Analysis tool (JSDA, version 1.2.27).

## Results

### Active ROM

**FIGURE 4:**
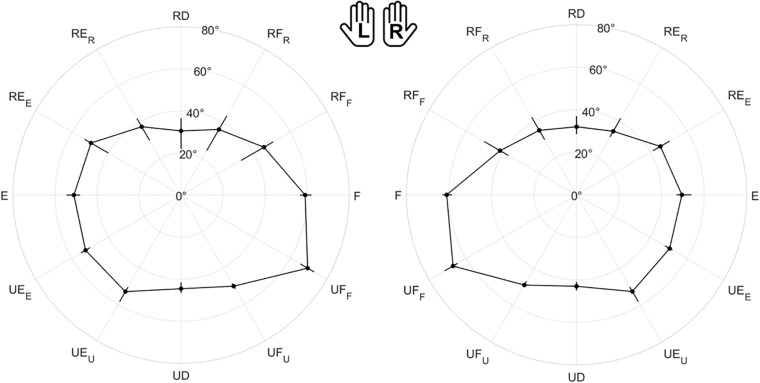
Evaluation of the Range of Motion (ROM) for all subjects. Left (L) and right (R) hands are represented on the corresponding side, mirrored with respect to the median dotted line. The median value of the maximum active ROM and the corresponding interquartile range (IQR) are shown in a polar plot, along 12 equally spaced directions. Since wrist movements are rotations, ROM is measured in degrees. The neutral position corresponds to 0° along each DoF.

Figure 4 shows the polar plot of the assessed ROM, namely the median value and the interquartile range between subjects along each direction. As shown by axes labels, hands are represented mirrored, with the purpose of highlighting between-hand symmetry. To visually help between-hand comparison, the reader can find a different representation of the same data in Supplementary Figure 1. Considering the 12 directions of movement, statistical analysis revealed a main effect of directions (χ^2^ = 348, *p* < 0.001), but no significant difference between hands (χ^2^ = 1.29, *p* = 0.256). These results are also important for the formulation of the JPM task, because the position of the JPM targets were normalized with respect to the evaluated ROM, setting a target distance equal to 80% of the assessed ROM.

### Joint Position Matching

**FIGURE 5:**
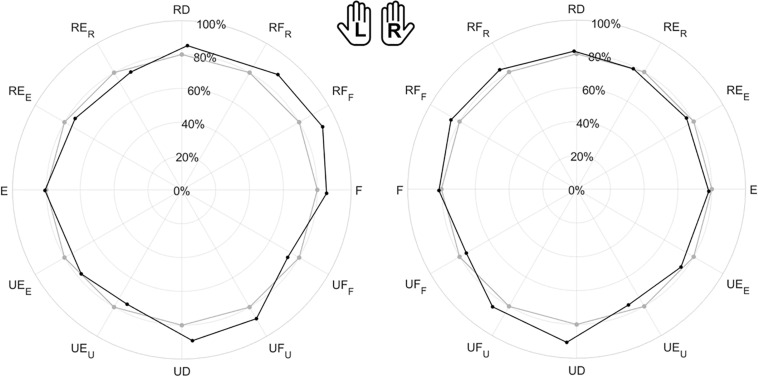
Evaluation of the Joint Position Sense (JPS) for all subjects. The polar plot shows the target position (gray points) and the mean matched positions (black points) obtained in the JPM task. Left (L) and right (R) hands are represented on the corresponding side of each panel, mirrored with respect to the median dotted line. The position of each point in the polar plot is determined by the mean angular *direction* and the mean extent of movement [*normalized magnitude* (ROM %)] between subjects.

Figure 5 is the overall polar representation of the assessed JPS, obtained by carrying out the JPM task for both hands. The radius was determined by the mean relative *normalized magnitude* of movements, i.e., the percentage extent of target or matching movements with respect to the ROM, while angular polar components are target or matched *directions* in degrees. This figure is the starting point for interpreting the statistical evaluations computed on outcome measures, particularly considering symmetry between hands and anisotropies among movement directions.

#### Left vs. Right Hand Comparison

**FIGURE 6:**
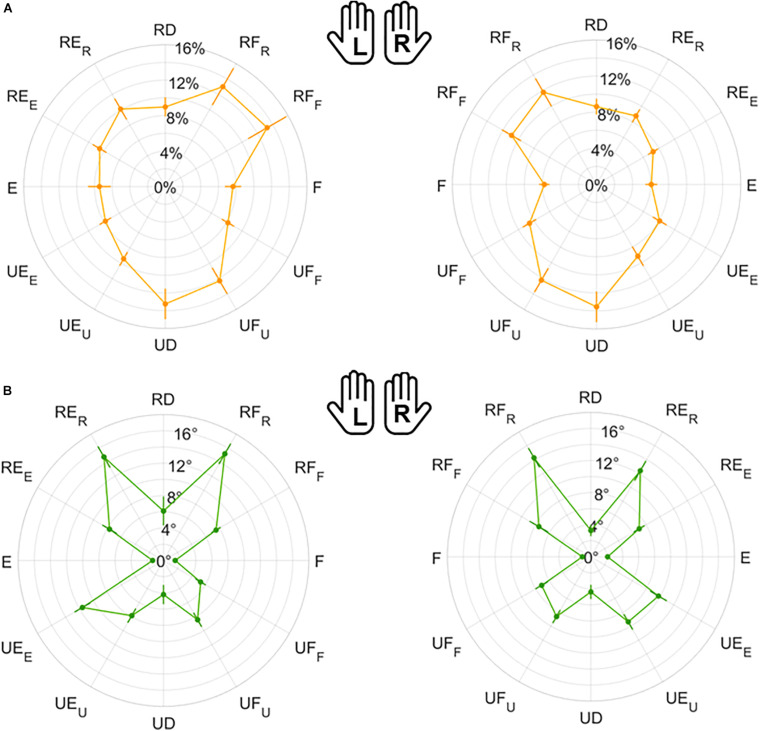
Mean results and standard error for Matching Error *normalized magnitude* **(A)** and *direction* **(B)**. Left (L) and right (R) hands are represented on the corresponding side of each panel, mirrored with respect to the median dotted line.

**FIGURE 7:**
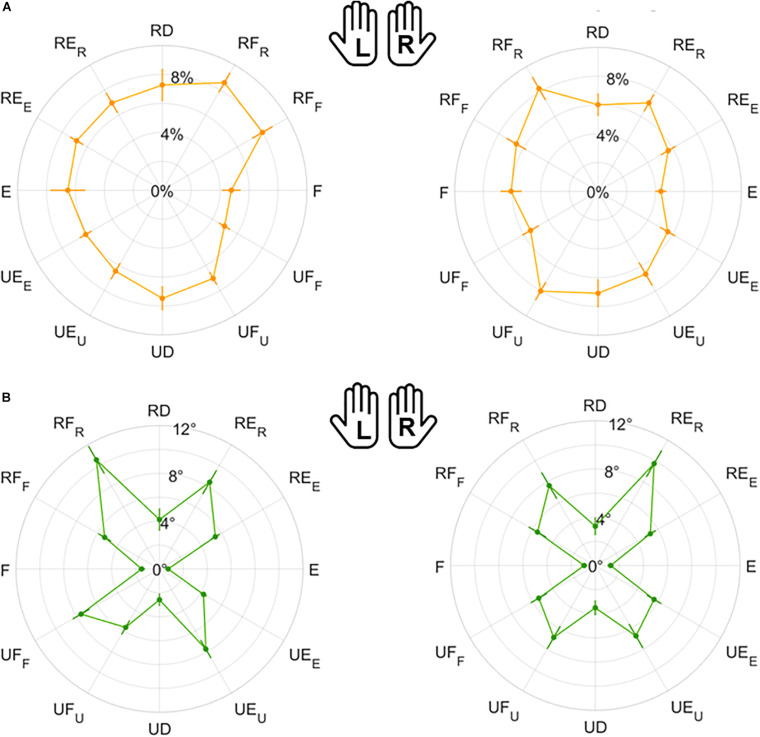
Mean results and standard error for Variability *normalized magnitude* **(A)** and *direction* **(B)**. Left (L) and right (R) hands are represented on the corresponding side of each panel, mirrored with respect to the median dotted line.

**FIGURE 8:**
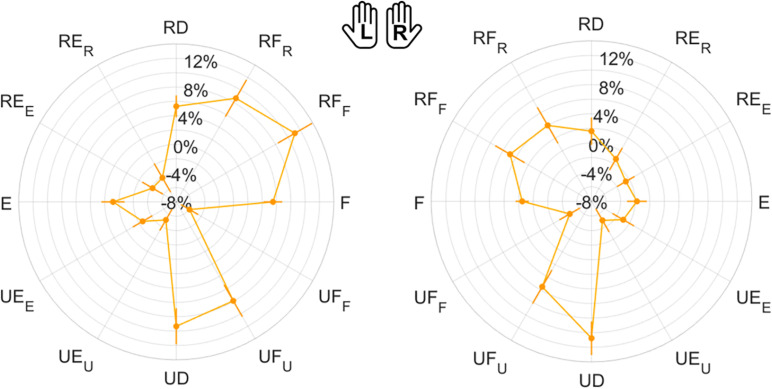
Mean results and standard error for Error Bias. Left (L) and right (R) hands are represented on the corresponding side of each panel, mirrored with respect to the median dotted line.

The visual comparison of the left (L) and right (R) sides of Figures 5–8 suggests that the performance of the two hands in the JPM task is similar. Additionally, to help the reader compare hand performance, Supplementary Figures 2–5 show the same data one on top of each other. The statistical analysis, summarized in Table 1, reports that no indicator presented significant differences between hands. Additionally, equivalence between hands was tested through TOST: since each indicator presented significant results for both lower and upper bound tests (Supplementary Table 1), JPM performance could be considered statistically equivalent between hands. Despite these results, we chose to perform the following statistical analysis considering left and right hand independently. This choice is explained by our interest in the statistical analysis of subsets of directions, whose differences among hands has not been considered as an objective of this work.

**TABLE 1:** Mean and median outcome parameters for each hand, and between-hand Kruskal-Wallis Test statistical results (χ^2^ and *p*-value).

**Hand**	**Mean**	**Median**	**χ^2^**	***p*-value**

**Matching error *normalized magnitude* (ROM %)**				
Right	9.2	7.9	1.28	0.258
Left	10.0	8.7		

**Matching error *direction* (°)**				

Right	7.2	6.0	0.08	0.777
Left	7.6	5.8		

**Variability *normalized magnitude* (ROM %)**				

Right	6.4	6.1	1.47	0.570
Left	6.8	6.1		

**Variability *direction* (°)**				

Right	5.2	4.1	0.32	0.570
Left	5.3	4.2		

**Error bias (ROM %)**				

Right	0.9	–0.2	1.76	0.185
Left	2.2	1.4		

#### 1-DoF Movements

Considering only FE and RUD planes (Table 2), ME, VAR, and EB had higher median and mean values along RUD with respect to FE in both hands, for both normalized *magnitude* and *direction* component. As shown in Table 2, these differences between planes were significant, except for VAR_mag_ in the right hand (χ^2^ = 1.56, *p* = 0.211). The analysis of directions always considered 1-DoF (RD/UD/F/E). With the exception of VAR_mag_, all indicators presented significant differences among directions in both hands (Table 3). Considering pairwise comparisons, EB was the only indicator that presented values along one direction, UD, that were significantly higher with respect to the other three directions, with differences always statistically significant at least in the right hand (Supplementary Table 2).

**TABLE 2:** Mean and median outcome parameters for FE and RUD planes (Flexion/Extension and Radial/Ulnar Deviation, 1-DoF planes) for each hand, and Kruskal-Wallis Test statistical results (χ^2^ and *p*-value) of 1-DoF planes comparison in each hand.

**Hand**	**Mean**		**Median**		**χ^2^**	***p*-value**
	**FE**	**RUD**	**FE**	**RUD**		

**Matching error *normalized magnitude* (ROM %)**						
Right	5.9	11.1	5.3	9.4	16.41	**<0.001**
Left	7.5	11.1	6.2	10.6	8.78	**0.003**

**Matching error *direction* (°)**						

Right	1.6	3.8	1.2	2.3	11.93	**<0.001**
Left	1.4	5.2	1.2	2.4	11.28	**<0.001**

**Variability *normalized magnitude* (ROM %)**						

Right	5.2	6.6	4.9	6.0	1.56	0.211
Left	57	7.4	5.0	6.6	4.24	**0.039**

**Variability *direction* (°)**						

Right	1.1	3.4	0.6	2.0	18.92	**<0.001**
Left	1.10	3.35	0.79	1.73	14.67	**<0.001**

**Error bias (ROM %)**						

Right	–0.1	6.2	–0.5	5.3	8.2	**0.004**
Left	3.2	7.3	3–0	7.9	4.0	**0.045**

*Bold text indicates a statistically significant difference (p-value < 0.05).*

**TABLE 3:** Kruskal-Wallis Test statistical results (χ^2^ and *p*-value) on 1-DoF (UD/RD/E/F) and on 2-DoF planes (UD/RD/E/F) in each hand.

**Hand**	**χ^2^**	***p*-value**	**χ^2^**	***p*-value**
	**1-DoF movements**		**2-DoF movements**	

**Matching error *normalized magnitude* (ROM %)**				
Right	18.69	**<0.001**	6.95	0.073
Left	12.18	**0.007**	4.89	0.180

**Matching error *direction* (°)**				

Right	21.12	**<0.001**	3.95	0.267
Left	13.39	**0.004**	15.05	**0.002**

**Variability *normalized magnitude* (ROM %)**				

Right	3.82	0.281	3.05	0.384
Left	5.21	0.157	10.72	**0.013**

**Variability *direction* (°)**				

Right	20.31	**<0.001**	3.02	0.389
Left	20.71	**<0.001**	2.03	0.567

**Error Bias (ROM %)**				

Right	17.65	**<0.001**	13.10	**0.005**
Left	10.10	**0.018**	40.40	**<0.001**

*Bold text indicates a statistically significant difference (p-value < 0.05).*

#### 2-DoF Movements

We inspected the presence of differences in the perception of 2-DoF combined directions (RF/RE/UE/UF). Contrarily from 1-DoF movements, here most indicators did not present significant differences among directions (Table 3). Additionally, also in 2-DoF movements, EB was the only indicator to show significant differences between directions in both hands. For this reason, we chose to highlight pairwise comparison results obtained for EB. As shown in Supplementary Table 3, EB emerged to be the only indicator that presented a direction, RF, significantly higher than the other three, with differences always statistically significant at least in the left hand.

#### 1-DoF vs. 2-DoF Movements

Finally, perception of 1-DoF and 2-DoF movements have been compared (Table 4): for both hands, Matching Error and Variability resulted significantly higher for 2-DoF movements, but only in their *direction* component (ME_dir_ and VAR_dir_). EB was positive in both hands (overshooting) in the case of 1-DoF matching movements. During 2-DoF, EB presented values significantly different from 1-DoF: EB, that is related to the *normalized magnitude*, resulted closer to the target position for 2-DoF matching movements, in both hands.

**TABLE 4:** Mean and median outcome parameters considering 1-DoF and 2-DoF directions for each hand, and Kruskal-Wallis Test statistical results (χ^2^ and *p*-value) of 1-DoF vs. 2-DoF comparison in each hand.

**Hand**	**mean**		**median**		**χ^2^**	***p*-value**
	**1 DoF**	**2 DoF**	**1 DoF**	**2 DoF**		

**Matching error *normalized magnitude* (ROM %)**						
Right	8.5	9.6	7.7	8.0	3.65	0.056
Left	9.3	10.3	8.0	8.8	1.22	0.269

**Matching error *direction* (°)**						

Right	2.7	9.5	1.6	8.1	101.14	**<0.001**
Left	3.2	9.8	1.5	8.4	97.29	**<0.001**

**Variability *normalized magnitude* (ROM %)**						

Right	5.9	6.7	5.1	6.3	3.42	0.064
Left	6.5	6.9	6.0	6.2	2.46	0.117

**Variability *direction* (°)**						

Right	2.2	6.6	1.3	5.0	78.56	**<0.001**
Left	2.2	6.9	1.1	5.6	88.14	**<0.001**

**Error bias (ROM %)**						

Right	3.0	–0.2	2.0	–1.7	7.29	**0.007**
Left	5.2	0.7	4.2	0.2	15.53	**<0.001**

*Bold text indicates a statistically significant difference (p-value < 0.05).*

## Discussion

This study investigated the accuracy of the proprioceptive representation of wrist position, in the space identified by FE and RUD movements. The chosen method was robot-based, with the purpose of providing reliable, quantitative data, to address the need of using an accurate tool for a repeatable and tailored proprioceptive assessment. The JPM task has been largely adopted in literature for mapping proprioceptive acuity in both healthy subjects and patients with sensorimotor dysfunctions (Squeri et al., 2011; Zabihhosseinian et al., 2015). Specifically, here accuracy and precision in the perception of direction and amplitude of wrist movements were evaluated along each assessed direction. Differently from previous works (Marini et al., 2016a,b), during matching movements, subjects were free to commit directional errors: for this reason, the two component of perception, extent and direction, could be evaluated independently. Another novelty of this work is the focus on mapping proprioceptive sensitivity of the wrist not only during single-DoF flexion/extension or ulnar/radial deviation movements, but also in coordinated 2-DoF movements. Actually, instead of four targets (F/E/RD/UD) (Marini et al., 2016b), 12 targets have been located along different equally spaced directions in the FE/RUD space. Moreover, along each direction, targets were placed at a specific distance: the wrist rotation required to reach the target corresponded to 80% of each subjects’ ROM measured along that direction. Considering muscle spindles and cutaneous afferents, we hypothesized that this method could lead to a comparable stretch of those receptors and consequently to similar resolutions. This hypothesis is supported by studies with microneurographic techniques, used to record activity from afferents, where the response of somatosensory receptors was found to change according to the distance from the limit of the joint rotation (Burke et al., 1988). In fact, given that at the farthest reachable point receptors find themselves at their maximum stretch, this method assures comparability both during different trials and between subjects. While the former is related to the anatomically different ROM found along different directions, the latter could be critical in a clinical setting, as wrist rigidity and loss of ROM characterize many clinical population that suffer from somatosensory deficits (Caligiuri, 1994; Luchetti et al., 2007; De Santis et al., 2015). Finally, the grip sensor was added to have a more controlled performance. Subjects were free to maintain a preferred grip, but the grip force was kept constant during the whole test. This assured that variability among trials was not due to changes in muscle activation related to a modified grip force applied to the handle (Barański and Kozupa, 2014) but reflected the intrinsic variability of the perceptual process.

### Between-Hands Symmetry of Perception

One main finding of this study concerns the presence of manual symmetries in proprioceptive perception: wrist position sense was comparable between the left hand and dominant right hand. Although these results were incongruent with some literature that assessed presence of asymmetries in the elbow joint (Goble and Brown, 2008), a symmetric perceptive performance was found in other works that assessed knee (Bullock-Saxton et al., 2001), arm (Carson et al., 1990), and the wrist joint (Adamo and Martin, 2009; Marini et al., 2016a). Despite dominant and non-dominant hands differing in terms of motor performance and dexterity, they resulted in comparable wrist sensory perception. Interestingly, adding new targets and DoF to the JPM task did not influence symmetry of perception (Marini et al., 2016a). Given evidence of site-specific symmetry of perception, these results are particularly crucial to evaluate JPS at specific joints (Han et al., 2013). Symmetry at the wrist could be essential information for clinical assessment: the unimpaired limb of a subject could act as a baseline for the evaluation and treatment of the impaired limb. In addition, presence of proprioceptive asymmetries in the wrist could serve as a marker for identifying the onset of some diseases (Miller-Patterson et al., 2018).

### Perceptual Anisotropy Over the Proprioceptive Space

#### Comparison With Previous Studies

Another main finding of this study is the anisotropy of wrist position sense, in both the perception of movement direction and extent. Particularly, our results should be compared with what has been found in previous works which investigated proprioception limited to FE and RUD directions in terms of extent of movement (Cappello et al., 2015; Marini et al., 2016a,b, 2017a). Our results suggest that error in the perception of movement extent is higher along the RUD than the FE plane. This finding seems to disagree with what has been found in previous work (Marini et al., 2016b). However, if we compute mean errors found in Marini et al. (2016b) as a percentage of the ROM considered in that work for target placements (80% of 20° for RUD, 80% of 40° for FE), the percentage of Matching Error would be higher along the RUD plane compared to FE (18.4% for RUD, 11.6% for FE). Moreover, our results are in accordance with another conclusion of that and other works (Janwantanakul et al., 2001), suggesting that proprioceptive accuracy becomes higher at the limits of the range of motion. Considering the target workspace of the present work, our targets were placed farther, in terms of degrees, with respect to those used in Marini et al. (2016b): accordingly, the errors in *normalized magnitude* reported in this study are lower than those found there. Regarding directional error, Ghez et al. (1995) demonstrated that, during reaching movements, variability and bias in the perception of direction were unaffected by distance. Nevertheless, a future study should investigate effects of target distance specifically on wrist perception of *direction*. Another future perspective could be the implementation of a global indicator able to point out the presence of wrist perceptive anisotropy and discriminate subjects with deficits from healthy ones.

#### Role of Cognition

Potential factors that could influence the JPM task are related to velocity and overall time duration. Particularly, the tau-effect (Goble, 2010), because of which faster movements are perceived as shorter, and this was avoided by keeping velocities constant among targets during the passive phase. This choice implied different time durations of movement for different target extents. Actually, the “cognitive” factor could affect the reliability of the JPS assessment, in the sense that the standard JPM paradigm implies a perceptual position memory of the tested subject. With patients this might be a problem and one might suggest to overcome the cognitive issue by using a bilateral version of the JMP task (Iandolo et al., 2015). However, this solution may not always be feasible in clinical practice, particularly in patients with severe impairments. In any case, making the JPM task as easy as possible, from the cognitive/attentional point of view, is critical for clinical applications. Although this study involved only healthy subjects, we did take into account the cognitive factor while choosing implementation details; for example, the break in the middle of the testing session was introduced in order to maintain the subjects cognitively focused on the task and avoid effects of both cognitive and muscle fatigue (Mugnosso et al., 2019).

#### Neuroanatomic Considerations

Further considerations should address the neuroanatomy of the wrist and, specifically, the density of mechanoreceptors and the level of innervation of wrist joint ligaments. In fact, immuno-histochemical studies of wrist ligaments show variations in the distributions of Ruffini and Pacini-like corpuscles, which contribute to proprioception (Hagert et al., 2005; Slutsky, 2005; Hagert, 2008). It has been found that ligaments with a higher level of innervation and density of mechanoreceptors are able to send signals during all wrist postures and motions, providing the sensorimotor foundation for the wrist (Hagert et al., 2005). Given these evidences, we hypothesized that stressing innervated ligaments with a different density of mechanoreceptors could lead to anisotropy in the identification of wrist position.

Some crucial features of proprioceptive information originate from muscle spindles, located in and oriented with muscle fibers. Although there are 14 muscles crossing the wrist joint, most of them contribute to wrist movements only as secondary function, while 5 are completely dedicated to wrist motions (Hoffman and Strick, 1999), including the palmaris longus, the flexor carpi radialis (FCR), the flexor carpi ulnaris (FCU), the extensor carpi radialis brevis and longus (ECR), and the extensor carpi ulnaris (ECU). Despite their names, Bawa et al. (2000) demonstrated that each of these muscles contributes to various wrist movements and is not activated in an isolated way. Since muscles can only pull and not push, wrist rotations also require antagonist muscles, pulling in the opposite direction of the agonist. In fact, each skeletal muscle has a unique pulling direction, determined by a line of action. Muscle pulling directions do not correspond with preferred directions, which are the directions along which the activation is highest. Additionally, muscles are more strongly recruited if movement directions significantly differ from their direction of pulling (Forman et al., 2020). For example, the FCR’s pulling direction was reported to be 28° away from pure wrist flexion, while FCU was reported to be 30° away, but toward ulnar deviation. Differently, pulling directions of ECU and ECR substantially deviate from pure extension (71° for ECU, 70° for ECR longus, and 48° for ECR brevis) (Bawa et al., 2000). Because of these differences, co-contraction was demonstrated to be higher along some directions, such as in flexion movements with respect to extension and in radial with respect to ulnar deviation (Bawa et al., 2000; Forman et al., 2020). Particularly, as shown by Fagg et al. (2002), wrist muscle pulling directions depend also on pronated, neutral or supinated posture of the forearm. We hypothesized that when pulling directions correspond to directions of movement the measure of stretch performed by muscle spindles in fibers results easier and more accurate. Additionally, greater amount of motor programming is involved when coordinating pulling actions of various muscles in the wrist joint (Forman et al., 2020). Likely, the simultaneous activation of many muscles as agonists during motion could be considered as a further source of noise. A future study using electromyography could clarify which muscle is active along each direction, how synergies influence perception and whether the level of grip force affected wrist position sense (Barański and Kozupa, 2014).

#### Coding Strategy

The last result presented shows a relation not only with the previously discussed factors that influence perception, but also with the strategy used to code proprioceptive information in this task. In fact, we obtained that combined movements, which involve simultaneously 2-DoF of the wrist, presented higher directional errors and variability with respect to those involving only 1-DoF. Moreover, errors and variability resulted comparable if we move to consider the extent of movements (*normalized magnitude*) instead of their *directions*. We postulate that the proposed task should have required a position coding to provide accurate information about wrist position. In position coding, the final equilibrium point is coded through the interpretation of body segments in the space, and matched, setting muscle length–tension parameters (Feldman, 1980). The alternative strategy involves the estimation of the relative position between the starting and target point, that is the so-called amplitude coding (Bock and Eckmiller, 1986; Ghez et al., 1995). While some works (Bock and Eckmiller, 1986; Marini et al., 2016c) found evidences supporting preference for amplitude coding of target positions, Marini et al. (2018) suggested a preference for joint position coding. Despite these apparently conflicting results, the crucial point is that the level of sensorimotor noise can induce the brain to switch among the different strategies (Körding and Wolpert, 2006). From our results, we could assume that subjects preferred amplitude coding for this task, rather than positional information. Errors in *normalized magnitude* were characterized by less anisotropy than those related to *directions*, as if, along some specific directions, subjects focused on the amplitude of movement, rather than on the final position reached. Particularly, anisotropy in directional error and variability was more evident when comparing 1-DoF movements with those where 2-DoF were involved and two vector components required to represent the space. We hypothesized that position resulted more difficult to be coded along directions requiring 2 DoF. Position coding is based on muscle length-tension parameters, directly measured by proprioceptors like muscle spindles. In this task, this measurement could be noisy and difficult to decode along 2-DoF combined directions of movement, because of the presence of multiple muscles activations, with different orientations and pulling directions, as discussed above. For this reason, strategy could have been switched online to amplitude coding, losing critical information about the direction of movement. This choice still resulted in an accurate discrimination of movement extent, but at the expense of higher directional errors along combined directions.

## Conclusion

In this work, we investigated how perception of wrist position changes across the space identified through multiple planes of flexion/extension and radial/ulnar deviation movements. We demonstrated the symmetry of wrist position sense between hands: this result confirmed previous findings related to other joints and a more restricted number of wrist movement directions. Interestingly, the same proprioceptive process was found to be characterized by anisotropy, that entails a different performance along different directions. Perception of the extent and direction of movements were different across the space. Particularly, reproduction of joint configurations that required the coordination of 2 DoF of the wrist was the hardest to code, thus leading to higher directional errors and variability as compared to 1-DoF matching movements.

## Data Availability Statement

The raw data supporting the conclusions of this article will be made available by the authors, without undue reservation, to any qualified researcher.

## Ethics Statement

The studies involving human participants were reviewed and approved by the Liguria Region (n.222REG2015). The patients/participants provided their written informed consent to participate in this study. Written informed consent was obtained from the individual(s) for the publication of any potentially identifiable images or data included in this article.

## Author Contributions

GA and JZ formulated the experimental question and design the study with FM and MH. GA collected the data, performed the data analysis and statistics, and wrote the manuscript. GA, MH, PM, and JZ participated in the results interpretation. JZ supervised the study and revised the final version of the manuscript.

## Conflict of Interest

FM was employed by company MathWorks. The remaining authors declare that the research was conducted in the absence of any commercial or financial relationships that could be construed as a potential conflict of interest.
