# Supplementary material for: Wrist Position Sense in Two Dimensions: Between-Hand Symmetry and Anisotropic Accuracy Across the Space
Source: Front Hum Neurosci. 2021 Apr 22;15:662768. doi: 10.3389/fnhum.2021.662768 (PMC8100524; doi:10.3389/fnhum.2021.662768)
Supplement: Supplementary file 1 [file Presentation_1.PDF]

Supplementary Material

1 Supplementary Figures

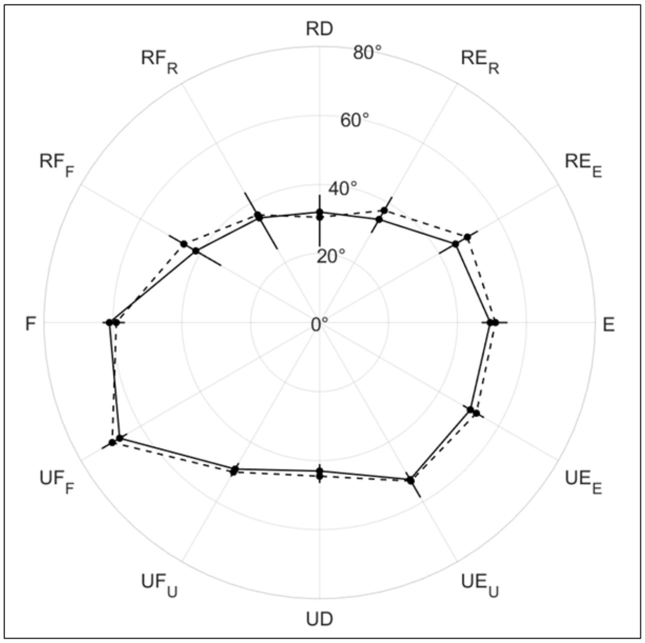

Supplementary Figure 1 Evaluation of the Range Of Motion (ROM) for the whole population of subjects. Left (L) and right (R) hands are represented by dotted and solid lines, respectively. The median value of the maximum active ROM and the corresponding interquartile range (IQR) are shown in a polar plot, along 12 equally spaced directions. Since wrist movements are rotations, ROM is measured in degrees. The neutral position corresponds to 0° along each DoF.

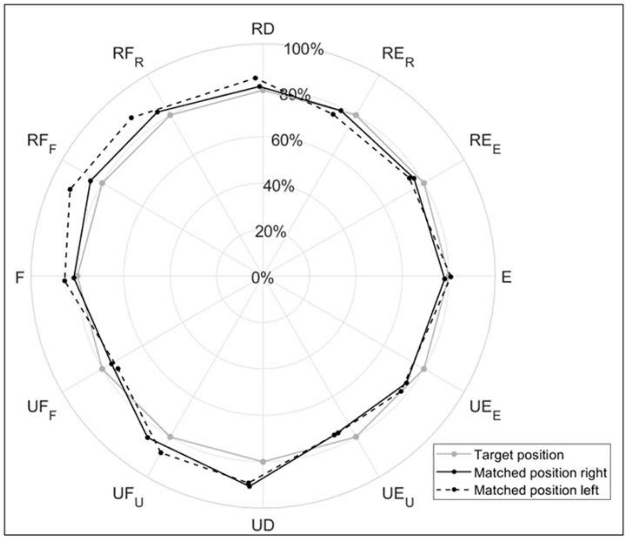

Supplementary Figure 2 Evaluation of the Joint Position Sense (JPS) for the whole population of subjects. The polar plot shows the target position (grey points) and the mean matched positions (black points) obtained in the JPM task. Left (L) and right (R) hands are represented by dotted and solid lines, respectively. The position of each point in the polar plot is determined by the mean angular *direction* and the mean extent of movement (*normalized magnitude* [ROM %]) between subjects.

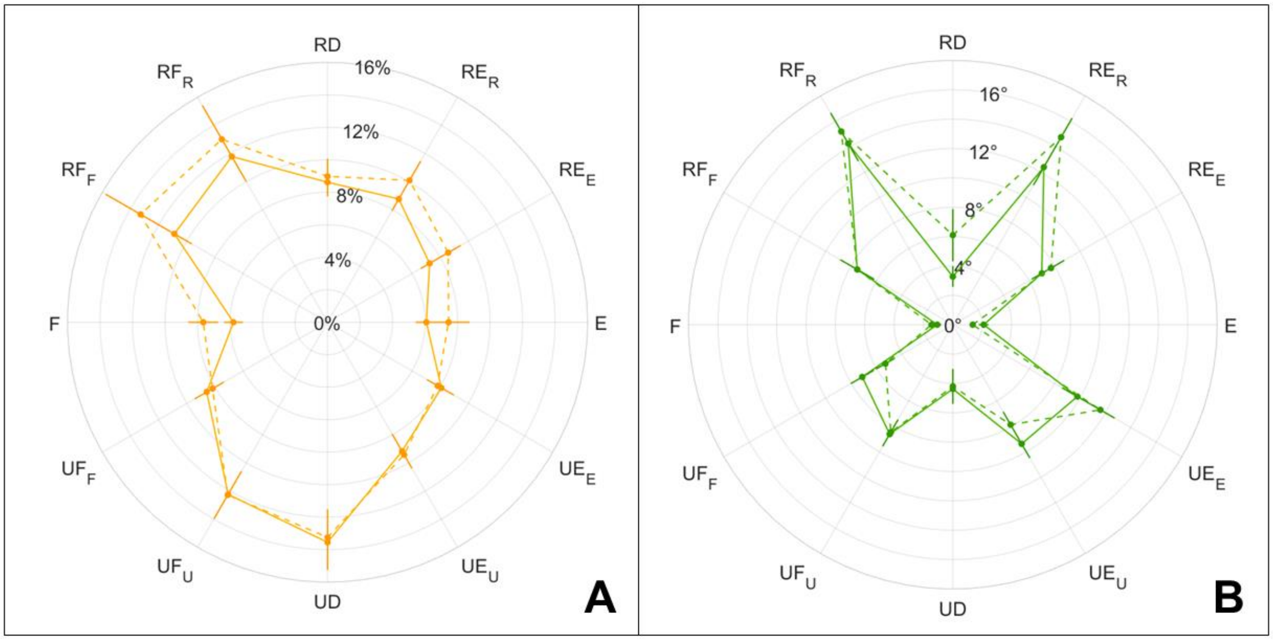

Supplementary Figure 3 Mean results and standard error for Matching Error *normalized magnitude* (A) and *direction* (B). Left (L) and right (R) hands are represented by dotted and solid lines, respectively.

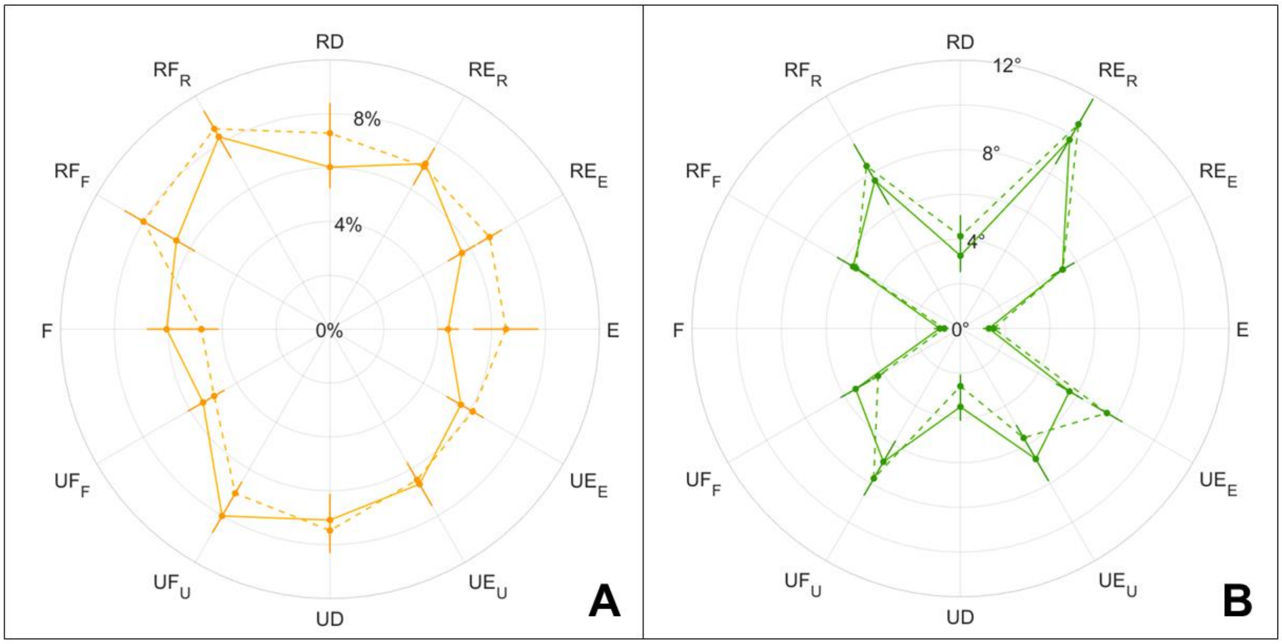

Supplementary Figure 4 Mean results and standard error for Variability *normalized magnitude* (A) and *direction* (B). Left (L) and right (R) hands are represented by dotted and solid lines, respectively.

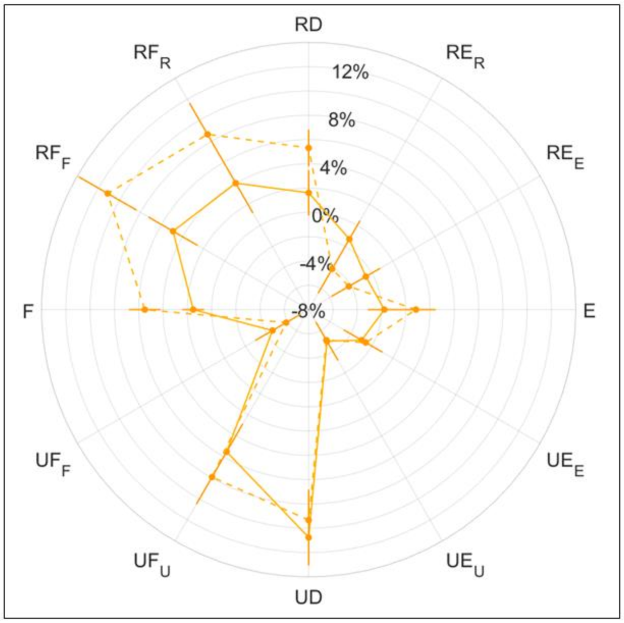

Supplementary Figure 5 Mean results and standard error for Error Bias. Left (L) and right (R) hands are represented by dotted and solid lines, respectively.

2 Supplementary Tables

	t	p-value
	Matching Error *normalized magnitude*	
TOST Upper	-3.04	**0.001**
TOST Lower	6.26	**< 0.001**
	Matching Error *direction*	
TOST Upper	-3.66	**< 0.001**
TOST Lower	5.63	**< 0.001**
	Variability *normalized magnitude*	
TOST Upper	-3.45	**< 0.001**
TOST Lower	5.84	**< 0.001**
	Variability *direction*	
TOST Upper	-4.18	**< 0.001**
TOST Lower	5.12	**< 0.001**
	Error Bias	
TOST Upper	-2.53	**0.006**
TOST Lower	6.77	**< 0.001**

Supplementary Table 1 TOST results to state between-hand equivalence (Cohen's d equivalence bounds Δ=[-0.5,0.5]) for each outcome indicator of the Joint Position Matching task. Right and left hand could be considered statistically equivalent when both upper and lower bound testing presented a significant p-value.

Hand	1-DoF direction	Error Bias mean [ROM %]	Error Bias median [ROM %]	W	p-value
RIGHT	UD	10.8	7.8		
	RD	1.6	-2.9	5.32	**< 0.001**
	E	-1.8	1.8	3.79	**0.037**
	F	1.5	1.6	4.13	**0.018**
LEFT	UD	9.3	10.4		
	RD	5.3	4.0	0.38	0.823
	E	0.8	0.5	3.86	**0.032**
	F	5.5	4.5	2.03	0.479

Supplementary Table 2 Mean and median Error Bias along each 1-DoF direction in each hand, and post-hoc pairwise comparisons (Dwass-Steel-Critchlow-Fligner Test, W (Wilcoxon rank sum test statistic) and p-value) between UD direction and RD/E/F.

Hand	1-DoF Direction	Error Bias mean [ROM %]	Error Bias median [ROM %]	W	p-value
RIGHT	RF	4.5	4.1		
	RE	-1.9	-2.0	3.85	**0.033**
	UF	0.47	-2.1	-2.50	0.288
	UE	-3.98	-4.0	-4.82	**0.004**
LEFT	RF	9.9	6.6		
	RE	-4.2	-6.0	8.03	**< 0.001**
	UF	1.0	0.6	-4.90	**0.003**
	UE	-3.9	-2.9	-7.76	**< 0.001**

Supplementary Table 3 Mean and median Error Bias along each 2-DoF direction in each hand, and post-hoc pairwise comparisons (Dwass-Steel-Critchlow-Fligner Test, W (Wilcoxon rank sum test statistic) and p-value) between RF direction and RE/UF/UE.
